# Eosinophils may serve as CEA-secreting cells for allergic bronchopulmonary aspergillosis (ABPA) patients

**DOI:** 10.1038/s41598-021-83470-z

**Published:** 2021-02-17

**Authors:** Yanfei Yang, qiqi Gao, Yangyi Jin, Mengdie Qi, Guohua Lu, HequanLi

**Affiliations:** 1 The First Affiliated Hospital of Zhejiang University School of Medicine, 79 Qingchun Road, Hangzhou, 310003 Zhejiang China; 2 Hangzhou TCM Hospital Affiliated to Zhejiang Chinese Medical University, Hangzhou, Zhejiang China; 3 Tongde Hospital of Zhejiang Province, Hangzhou, Zhejiang China

**Keywords:** Respiratory tract diseases, Respiratory signs and symptoms, Biomarkers

## Abstract

Allergic bronchopulmonary aspergillosis (ABPA) is a condition characterized by an exaggerated response of the immune system to the fungus Aspergillus. This study aimed to assess the relationship between carcinoembryonic antigen (CEA) and eosinophils in ABPA patients. We describes a case of a 50-year-old patient who was diagnosed with ABPA presenting with high level of CEA and eosinophils. Besides,we used immunohistochemistry and immunofluorescence to identify eosinophils and CEA in sections which were obtained by Endobronchial ultrasound-guided transbronchial lung biopsy aspiration (EBUS-TBLB). The sections were then visualized using confocal microscopy. We also retrospectively analyzed a cohort of 37 ABPA patients between January 2013 and December 2019 in our hospital*.* We found the patient whose serum CEA levels were consistent with eosinophils during the follow-up (r = 0.929, *P* = 0.022). The positive expression of CEA and abnormal expression of eosinophils was higher in the ABPA tissue compared to the normal lung tissue. The co-localization was represented as pixels containing both red and green color in the image (with various shades of orange and yellow) which signified that eosinophils were immunohistochemically positive for CEA. Patients with higher levels of eosinophils had higher levels of CEA in the serum (*P* < 0.001). The results of Pearson correlation analysis showed that the levels of eosinophils were positively correlated with serum CEA levels (r = 0.459 and r = 0.506, *P* = 0.004 and *P* = 0.001). Serum CEA level is elevated in ABPA patients. The elevated serum CEA level was shown to be normalized after treatment. Increased CEA levels in ABPA patients may be positively correlated with eosinophil levels, and eosinophils may be served as CEA-secreting cells in patients with ABPA.

## Introduction

Allergic bronchopulmonary aspergillosis (ABPA) is characterized by an allergic inflammatory response to *Aspergillus fumigatus* colonized in the trachea^1^. It is manifested as poorly controlled asthma and affected approximately 4 million patients worldwide^2^. Some patients may even suffer from irreversible airway obstruction and pulmonary fibrosis^3^. Besides, a significant eosinophil infiltration can be seen in the pathological specimens of ABPA patients^4^.

Carcinoembryonic antigen (CEA) was initially discovered as a tumor antigen^5^. Serum CEA level is typically identified as a candidate biomarker for tumor progression^6^. Therefore,people with high levels of serum CEA usually indicates the presence of malignant tumors. However, Japanese scholars have reported 13 patients with ABPA, in which 7 of them had elevated levels of CEA in the peripheral blood. In case of pulmonary consolidation, the serum CEA levels were gradually returned to normal level^7^. Regrettably, the study did not mention the precise source of CEA in ABPA patients. Recently, CEA has also been proposed as a clinical marker, reflecting the activity of acquired idiopathic generalized anhidrosis (AIGA)^8^. Immunohistochemistry of 10 AIGA cases showed increased levels of CEA in the eccrine sweat glands^9^. A previous study has pointed out that neutrophil extracellular traps (NETs) are associated with CEA^10^. That means CEA is no longer just a tumor marker.

Thus, aim of the study was to assess whether eosinophils of allergic bronchopulmonary aspergillosis (ABPA) patients express carcinoembryonic antigen (CEA). The first part of the manuscript describes a case study of a patient with high levels of CEA with a suspected diagnosis of ABPA, the second part describes a retrospective cohort study of 37 hospitalized ABPA patients.

To the best of our knowledge, the presented study is the first to report the relationship between serum CEA level and eosinophils in patients with ABPA.

## Materials and methods

This research study was reviewed and approved by Research Ethics Committee of The First Affiliated Hospital,College of Medicine, Zhejiang University with informed consent obtained from all participants prior to the start of the study.All methods were carried out in accordance with relevant guidelines and regulations.

### Study design

The pathological tissue was obtained by Endobronchial ultrasound-guided transbronchial lung biopsy aspiration (EBUS-TBLB) from a 50-year-old patient who was diagnosed with ABPA, presenting with high level of CEA and eosinophils. We used immunohistochemistry and immunofluorescence to identify eosinophils and CEA in sections gained by EBUS-TBLB and tracked the clinical data of this ABPA patient.

Also, a total of 37 patients were included in the study. The clinical details of all patients were collected from themedical records, including demographic data, pulmonary comorbidities, clinical symptom and sign, laboratory examinations, thoracic CT findings, diagnosis. We analyzed the elevated levels of CEA and peripheral blood eosinophils in the case of ABPA patien and then, following the initial case, we hve performed a retrospective study analyzing a cohort of 37 ABPA-diagnosed patients, comparing their CEA and eosinophil count, relating both parameters in this disease.

### Materials

The levels of *Aspergillus fumigatus*-specific IgG and *Aspergillus fumigatus*-specific IgE were detected by using a commercial enzyme-linked immunosorbent assay (ELISA) kit (Thermo Fisher Scientific, Waltham, MA, USA). Besides, interleukin-5 (IL-5) was detected by using human IL-5 platinum ELISA kit (eBioscience Inc., San Diego, CA, USA). Blood eosinophils were measured using a routine blood testing kit. Eosinophils were detected by myelin basic protein (MBP) antibody (Sangon Biotech Co., Ltd., Shanghai, China) and hematoxylin and eosin (H&E) staining. CEA was detceted by CEA monoclonal antibody (Thermo Fisher Scientific, Waltham, MA, USA). Anti-rabbit IgG secondary antibodies conjugated with Alexa-Fluor 488 and anti-mouse IgG secondary antibodies conjugated with Alexa-Fluor 594 were used.

### Immunohistochemistry and immunofluorescence

The sections were de-waxed, rehydrated, and incubated with 3% hydrogen peroxide in methanol to block endogenous peroxidase activity at room temperature for 10 min^11^. For antigen retrieval, deparaffinized and rehydrated sample tissue sections were pretreated by microwave irradiation for 20–30 min in 0.01 mol/l citrate-buffered saline (pH 6.0)^12^.

Closed non-specific binding was performed, and normal goat serum was then added to the sections. Sections were then incubated for overnight at 4 °C with primary antibodies (mouse anti-human CEA and rabbit anti-human MBP antibodies) diluted in blocking solution. Membranes were incubated with a secondary antibody, and then goat anti-mouse/rabbit antibody, and diluted at an appropriate dilution in 1% bovine serum albumin (BSA) for 2 h at room temperature. Diaminobenzidine (DAB) color reactions were visualized under a microscope (5–10 min). After rinsing thricewith phosphate-buffered saline (PBS), staining was carried out by DAB before counterstaining with Hematoxylin^13^.

Double-immunofluorescence staining was performed as described previously^14^. In brief, the sections were analyzed via antigen retrieval, blocked for endogenous peroxidase and non-specific epitopes, and incubated with primary antibody at 4 °C overnight. Antibody detection was performed using secondary antibodies (Anti-rabbit IgG secondary antibodies conjugated with Alexa-Fluor 488 and anti-mouse IgG secondary antibodies conjugated with Alexa-Fluor 594). Samples were mounted in 4′,6-diamidino-2-phenylindole (DAPI), and visualized under a LSM880 Meta laser-scanning confocal microscope (Carl Zeiss).

### Inclusion criteria

The diagnosis of ABPA is currently done by combining clinical, radiological, and immunological findings based on the criteria proposed by The International Society for Human and Animal Mycology (ISHAM) in 2013 (see Table 1 which was quoted from a review article by Agarwal et al.^15^). The ISHAM proposed new diagnostic criteria in 2013, which define asthma or cystic fibrosis as predisposing conditions and included 2 obligatory criteria: (1) immediate cutaneous hypersensitivity to *Aspergillus* antigen or elevated IgE levels against *A fumigatus*, and (2) elevated total IgE levels. They also included 3 minor criteria, at least 2 of which should be satisfied for ABPA diagnosis, namely: (1) presence of precipitating or IgG antibodies to *A fumigatus*, (2) radiographic features in the lungs consistent with ABPA, and (3) peripheral blood eosinophilia^16^.

**Table 1:** Newly proposed diagnostic criteria for allergic bronchopulmonary aspergillosis.

Predisposing conditions	
	Bronchial asthma, cystic fibrosis ,et al
Obligatory criteria (both should be present)	
	(1) Type I *Aspergillus* skin test positive (immediate cutaneous hypersensitivity to *Aspergillus* antigen) or elevated IgE levels against *Aspergillus fumigatus*
	(2) Elevated total IgE levels (> 1000 IU/mL)^*a*^
Other criteria (at least two of three)	
	(1) Presence of precipitating or IgG antibodies against *A *. *fumigatus* in serum (2) Radiographic pulmonary opacities consistent with ABPA^*b*^ (3) Total eosinophil count > 500 cells/μL in steroid naïve patients (may be historical)

^a^If the patient meets all other criteria, an IgE value < 1000 IU/mL may be acceptable.

^b^The chest radiographic features consistent with ABPA may be transient (i.e. consolidation, nodules, tram‐track opacities, toothpaste/finger‐in‐glove opacities, fleeting opacities) or permanent (i.e. parallel line and ring shadows, bronchiectasis and pleuropulmonary fibrosis).

### Exclusion criteria

We have excluded out patients with parasitic diseases or suffered from parasitic diseases previously. Patients with current or past tumor history are also included in our exclusion criteria. At the same time, we reject patients whose immune system were impaired. Patients who are using immunosuppressive drugs are not included in our research,either.

### Statistical analysis

All data were presented as *M*(*P*25, *P*75), and were analyzed using SPSS 25.0 software (IBM, Armonk, NY, USA)*.* *P* ≤ 0.05 was considered statistically significant, and *P* ≤ 0.01 as highly significant. Pearson correlation coefficient lower than 0.4 indicates poor reproducibility, which range from 0.4–0.75, denoting fair reproducibility, and equal to 0.75 indicates good reproducibility^17^. (*M* represents the median of the data).

### Ethics approval and consent to participate

The study was approved by Research Ethics Committee of The First Affiliated Hospital,College of Medicine, Zhejiang University (Reference Number:2019/196). Ethics approval and consent to participate have been uploaded.

## Results

### A patient with high level of CEA was diagnosed as ABPA

**Figure 1:**
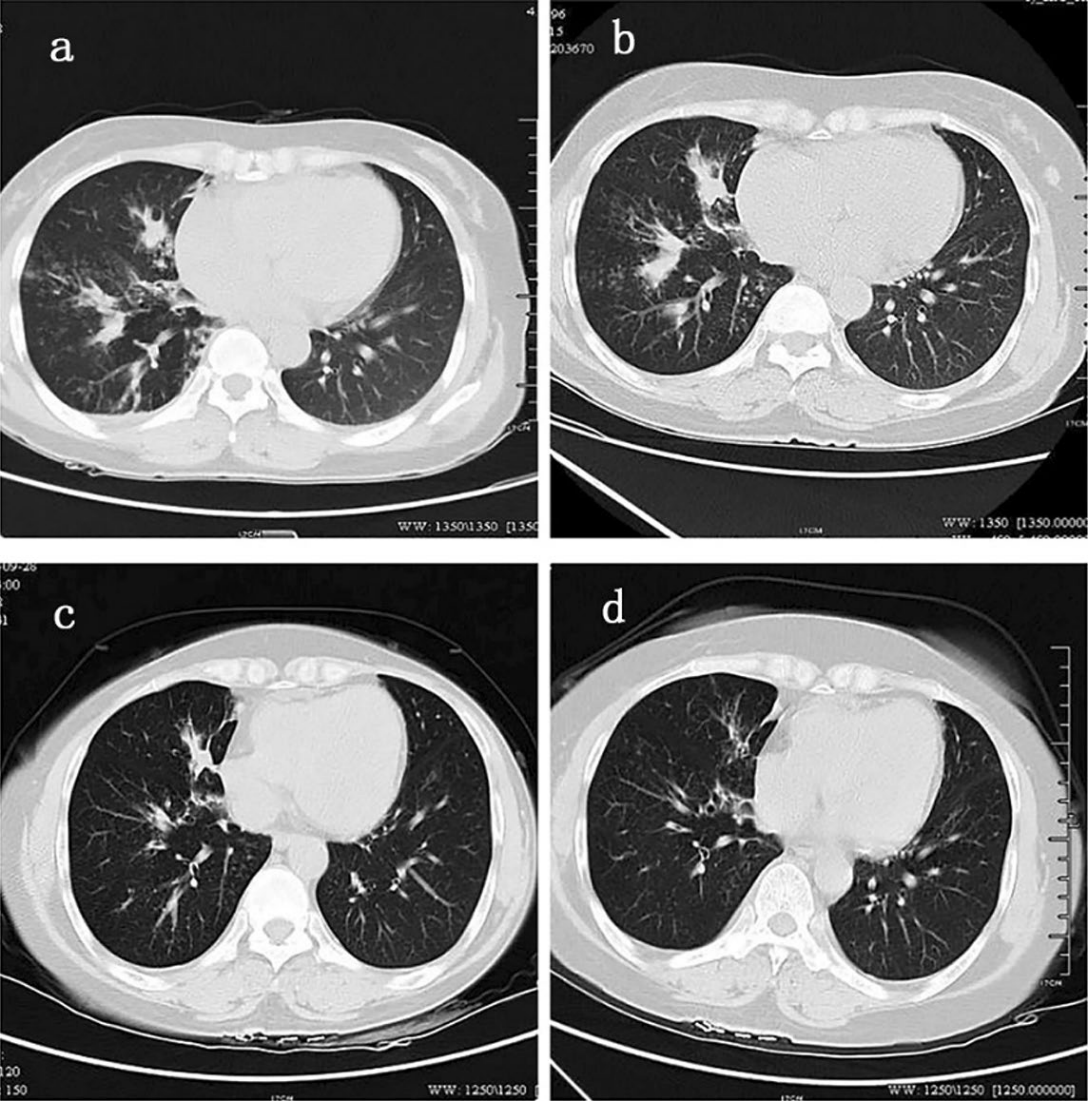
Patient underwent follow-up CT. (**a**) When this patient first came to our hospital, CT appeared as exudation with consolidation. (**b**–**d**) After treatment with glucocorticoids, the pulmonary consolidation significantly reduced.

A 50-year-old female patient suffering from cough and dyspnea for 2 years was diagnosed with lung cancer because of high serum levels of CEA and lymphadenopathy before she was admitted to our hospital. But the result of positron emission tomography-computed tomography (PET-CT) examination and bone marrow biopsy showed no evidence of tumor. Initial laboratory tests after admission showed elevated CEA level (38.0 ng/mL, reference range < 5.2 ng/mL) and a high count of eosinophils (5700/μL). CT of the lung revealed bronchiectasis and infiltration of the right middle and right lower lobes (Fig. 1a). EBUS-TBLB were employed to evaluate mediastinal lymph nodes and lung lesions, showing accumulation of eosinophils in the lungs and lymph nodes. According to the proposed diagnostic criteria for ABPA, the patient was diagnosed with ABPA based on the history of bronchial asthma, as well as the elevated levels of *Aspergillus fumigatus*-specific IgG,serum total IgE and *Aspergillus fumigatus*-specific IgE. Systemic corticosteroid therapy (prednisone 40 mg/day) was initiated, and the patient’s symptoms were dissipated. CT scan of the chest indicated disappearance of pulmonary infiltrates and mucoid impaction (Fig. 1a-d) after 6 months.

### Eosinophils and CEA were decreased simultaneously

**Figure 2:**
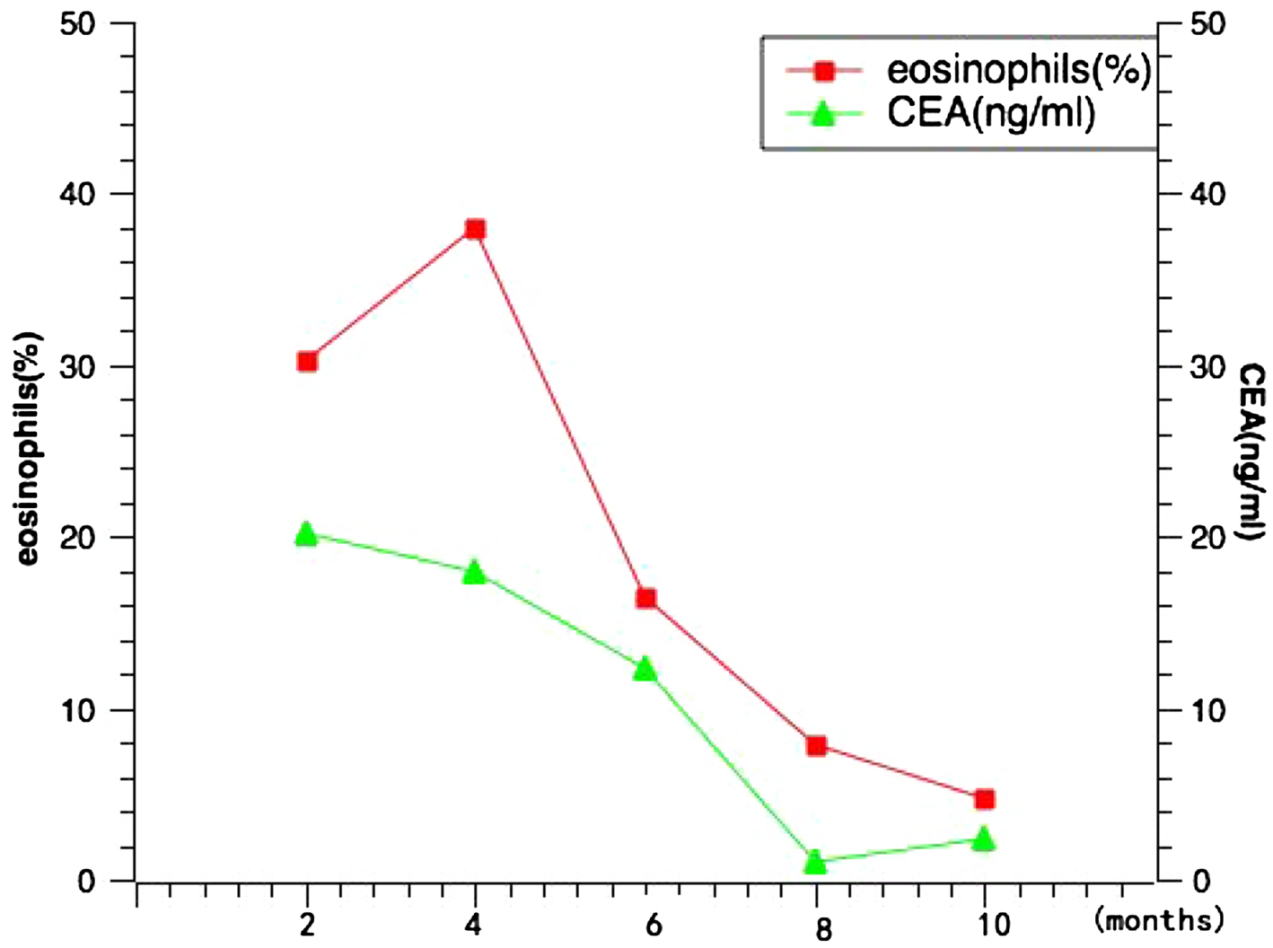
The relationship between CEA and eosinophils in this patient.

As she was diagnosed with ABPA, she has received glucocorticoid therapy. After treatment, the patient’s symptoms were remarkably improved. During the follow- up, she performed to measure CEA and eosinophils every month or so and adjust her hormone dose at the same time. The decreased serum CEA levels were consistent with eosinophils during the follow-up (Fig. 2). The correlation between the serum CEA levels and eosinophil levels in the 6-month follow-up duration was analyzed by using Pearson correlation analysis, wherein significant correlations were observed (Table 2, r = 0.929, *P* = 0.022).

**Table 2:** The relationship between CEA and eosinophils in this person.

	Median (range)	Correlation coefficient	*P*
Eosinophils (%)	8.81 (1.65, 19.10)	0.929	**0.022**
CEA (ng/ml)	14.31 (6.30, 34.10)		

### Activated CEA was partially co-localized on eosinophils

**Figure 3:**
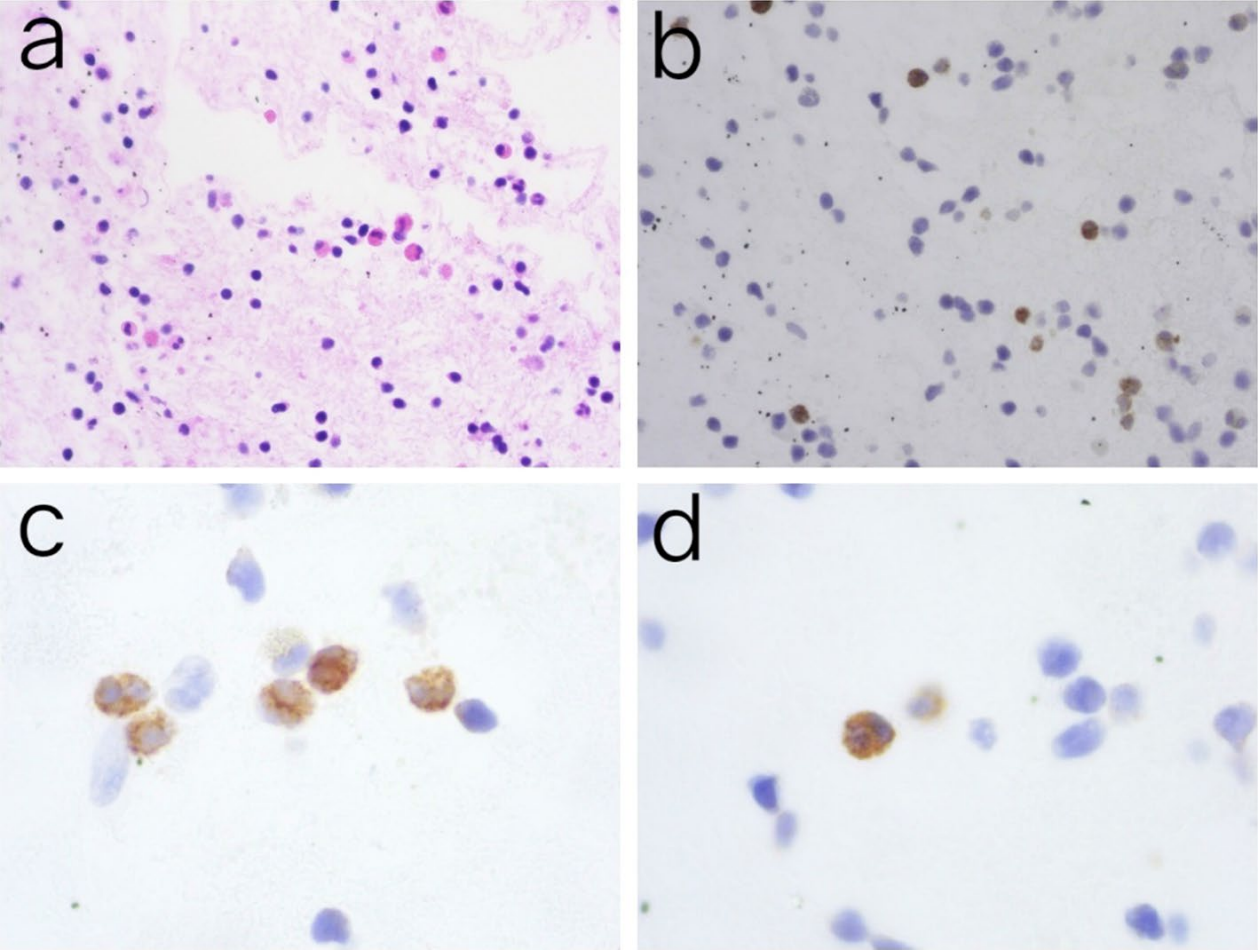
Lung tissue stained with H&E and immunohistochemistry. (**a**) lung tissue stained with H&E, Alveolar was infiltrated with eosinophils (× 400 magnification). (**b**) There are CEA positive cells in lung tissue (× 400 magnification). (**c** and **d**) Immunostaining for CEA showed that CEA was observed in the cells which were characteristic of eosinophils (× 800 magnification).

**Figure 4:**
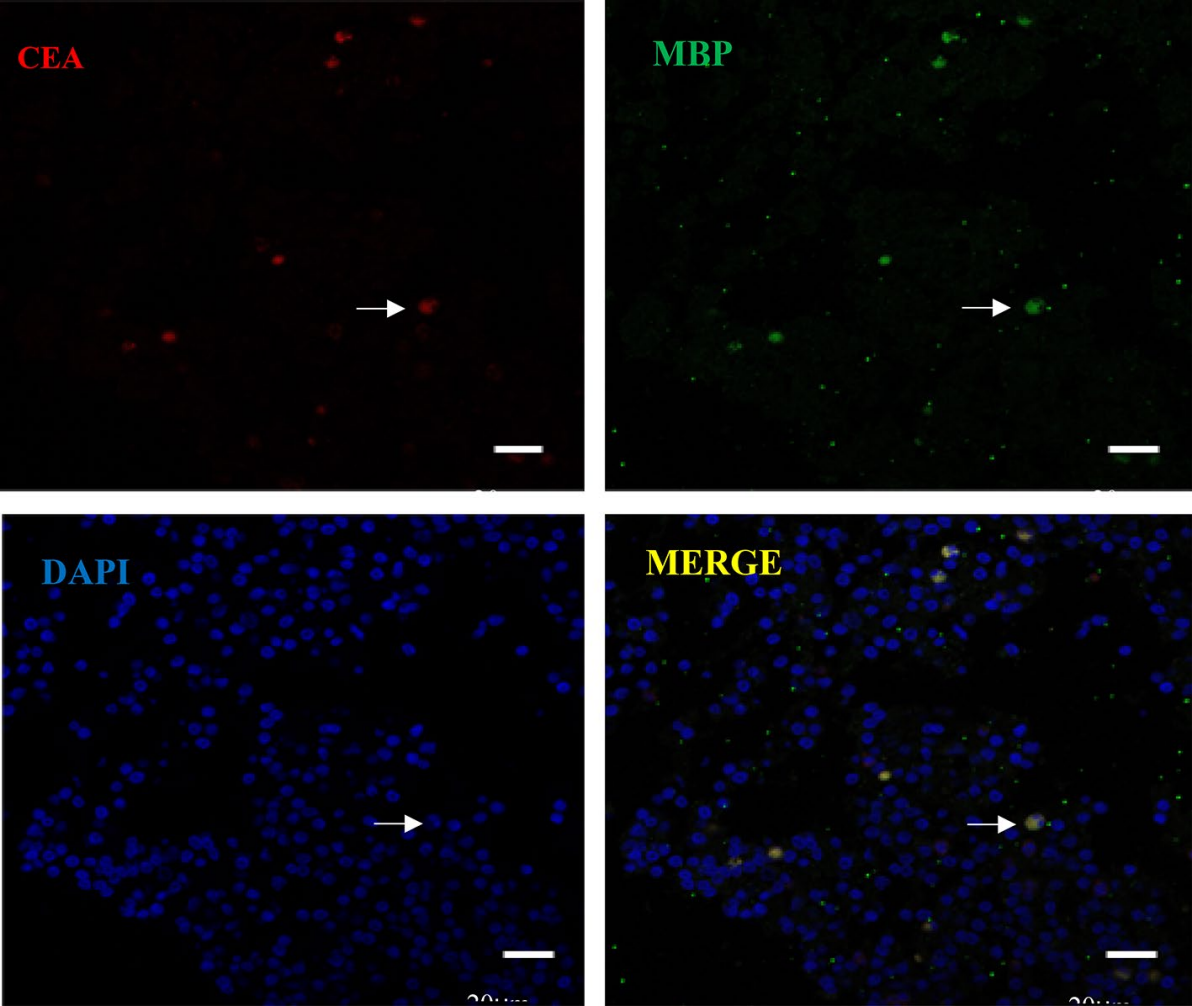
Lung section with a MBP–positive cell colabeled with CEA antigen localized on Cytoplasm, as indicated by the arrow. Scale bars = 20 μm.

**Figure 5:**
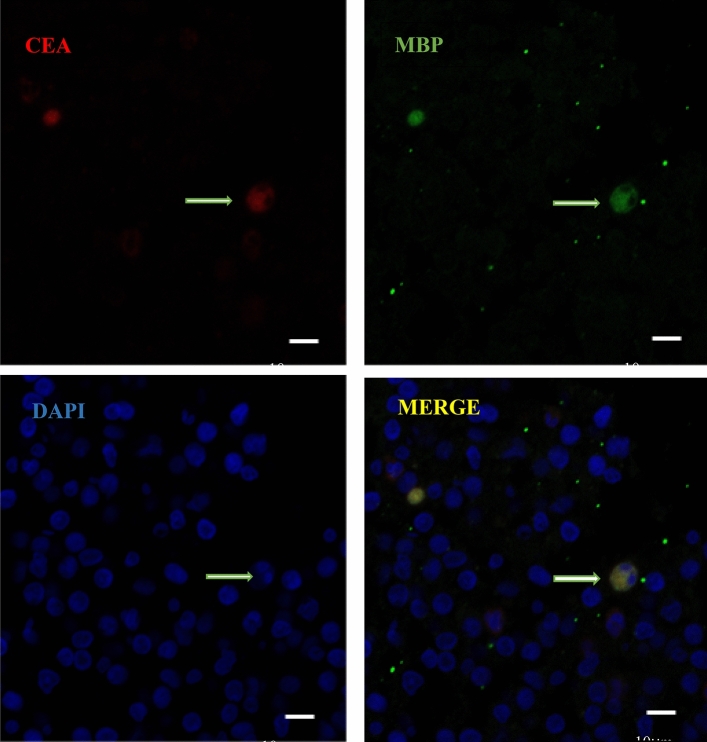
Lung section with a MBP–positive cell colabeled with CEA antigen localized on Cytoplasm, as indicated by the arrow. Scale bars = 10 μm. Dual immunofluorescence was performed using the eosinophils marker MBP (green) and the CEA Monoclonal Antibody (RED). Nuclei stained with DAPI (blue).Both double-labeling immunofluorescence assays were performed in lung section because of their positive induced in eosinophils . DAPI, 4′,6′-diamino-2-phenylindole; MBP (also called Anti-PRG2 rabbit polyclonal antibody) eosinophil major basic protein ; CEA (CEA Monoclonal Antibody),Carcino Embryonic Antigen.

Initially, H&E staining confirmed that a lot of eosinophils in the lung (Fig. 3a). CEA was first detected by immunocytochemistry.The yellow–brown granules observed in the cells indicated a positive result for CEA. Additionally, a great number of CEA markers were found in the cells (Fig. 3b). The results revealed that the eosinophils were immunohistochemically positive for CEA under high magnification (Fig. 3c,d). Immunofluorescence analysis also unveiled that eosinophils ,as well as CEA, were identified in the lung. Moreover, Lung section with a MBP–positive cells were colabeled with CEA antigen which was localized on Cytoplasm (Figs. 4, 5).

### Increased serum CEA levels were positively associated with eosinophil levels

**Figure 6:**
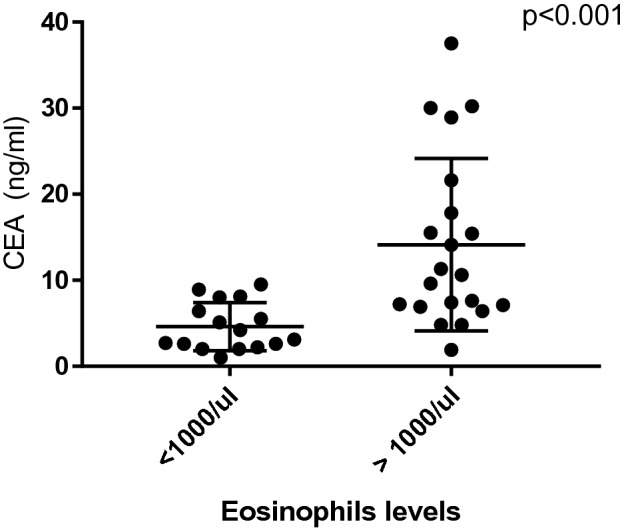
A higher eosinophil count is with the echo of higher levels of CEA.

**Figure 7:**
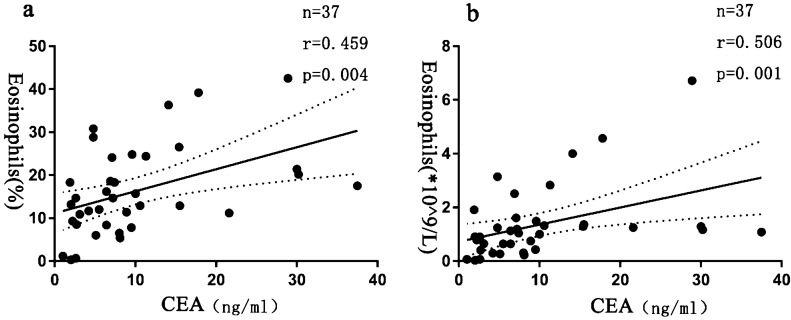
Correlation of CEA and EOS in ABPA patients. (**a**) Relationships among CEA and EOS%. (**b**) Relationships among CEA and EOS counts.

Clinical data of the patients are listed in Table 3. Twenty four male and 13 female patients were included in the current study. Before commencing the treatment, 25 (25/37) patients had serum CEA levels higher than normal. As shown in Table 4, the serum CEA levels were significantly increased, and the mean serum CEA level was markedly higher in ABPA patients than that in healthy subjects (As expected on healthy subjects, the CEA content was low which was below 5.05 ng/ml.). Moreover, 37 patients were assigned to two groups based on peripheral blood eosinophil counts. Independent -sample t-test was employed to compare serum CEA levels between the two groups. There are 16 patients whose eosinophils were less than 1000/μl, and 21 patients had eosinophils level which was higher than 1000/μl.Clnical data were similar in patients comparing to patients whose eosinophils were higher than 1000/μl or not,except for erythrocyte sedimentation Rate (ESR) (see Table 5). The results showed that patients with higher eosinophil levels had higher serum levels of CEA (*P* < 0.001, Fig. 6). Besides, we noted that the eosinophil levels were positively correlated with serum CEA levels (r = 0.459 and r = 0.506, *P* = 0.004 and *P* = 0.001) by Pearson correlation coefficient (Fig. 7).

**Table 3:** Demographics and clinical features of 37 patients with ABPA (n = 37).

Clinical parameters	Number (proportion)
Age (years)	55.00 ± 15.96
Gender (male/female)	24 (64.86%) /13 (35.14%)
**Symptom**	
Cough	37 (100.00%)
Expectoration	29 (78.38%)
Hemoptysis	7 (18.92%)
Short of breath	20 (54.05%)
Heat	5 (13.51%)
**Comorbidities**	
Asthma	13 (35.14%)
COPD	9 (24.32%)
Interstitial lung disease	4 (10.81%)
Bronchiectasis	28 (75.68%)
**Treatment**	
Voriconazole	9 (24.32%)
Voriconazole and glucocorticoid	14 (37.84%)
Glucocorticoid	4 (10.81%)
Itraconazole and glucocorticoid	9 (24.32%)
Itraconazole	1 (2.70%)
**Ending**	
Relief or (and) stability	21 (56.76%)
Relapse or (and) progression	16 (43.24%)

**Table 4:** Laboratory Characteristics of ABPA patients.

Laboratory results	Normal range	people (n = 37)
Eosinophils (/uL)	20–500 (/uL)	1080 (540,1430)
Eosinophils (%)	0.5–5.0 (%)	14.70 (8.90,22.75)
TIgE (KU/L)	0.0–100.0(KU/L)	
< 5000		28 (75.68%)
≥ 5000		9 (14.32%)
sIgE (kU/L)	0.00–0.35(KU/L)	5.69 (2.83,12.02)
ESR (mm/h)		14.00 (7.00,33.00)
CRP (mg/L)		6.76 (2.48,13.83)
CEA (ng/ml)	0.00–5.05(ng/ml)	7.20 (3.65,12.70)
CEA < 5.00		12 (32.43%)
5 ≤ CEA < 10		14 (37.84%)
≥ 10.00		11(29.73%)

**Table 5:** Basic information of the two groups of ABPA patients.

Parameter	> 1000/ul	< 1000/ul	*P* value
Age	51.38 ± 16.94	59.75 ± 13.64	0.483
Gende (male/female)	13/8	11/5	0.839
CEA (ng/ml)	10.60 (7.00,19.70)	3.65 (2.30,7.60)	**0.001**
sIgE (kU/L)	5.97 (3.12,14.48)	5.09 (1.74,16.48)	0.674
ESR (mm/h)	18.0 (5.25,41.5)	13.00 (7.50,22.00)	**0.01**
CRP (mg/L)	8.30 (2.71,14.37)	4.90 (2.48,7.53)	0.584

## Discussion

ABPA is the most significant manifestation of allergic aspergillosis that occurs worldwide, but much attention has not been paid by the scholars^18^. Meanwhile, eosinophils promote inflammation, and are known to play a beneficial role in isolating and controlling a disease site^19^. To our knowledge, CEA is the most commonly used serum marker in the management of breast cancer, and its expression showed correlation with clinicopathological characteristics of gastric carcinoma^20^. CEA was first identified as colon cancer antigen in 1965. The serum CEA levels were higher in patients with colon cancer when compared to healthy individuals, and this led to its clinical application as a diagnostic biomarker for colorectal cancer^21^. Recently, the serum levels of CEA and the trend of its changes in the treatment process have been previously validated^22^. Thus, some patients with elevated CEA levels were easily misdiagnosed as lung cancer. In the current study, CEA levels were found to be significantly higher in ABPA patients compared with the normal population*.* The comparative data demonstrated the necessity of assessment of relationships between the eosinophils and serum CEA level in ABPA patients. In the current study, the serum CEA level was shown to be decreased after treatment, indicating that glucocorticoid therapy might have inhibitory effects on CEA level in ABPA patients. The serum CEA level was found to be positively correlated with blood eosinophils. Previous studies have shown that elevated serum CEA levels in patients with ABPA are associated with consolidation of lungs, mucus plugs, and localized inflammation of lungs^7^. Several scholars have pointed out that serum concentrations of CEA were significantly increased in asthmatic patients with mucoid impactions when compared to those patients without mucoid impactions or patients with bronchiectasis^23^. However, in our research, the serum levels of CEA were found to be associated with eosinophil count. This is also the first report to present an ABPA case with the relationship between serum CEA level and eosinophils count, and our findings may be significant for clinicians. According to our study, patients with elevated serum CEA levels were diagnosed with benign diseases (ABPA). Compared with normal people, the mean serum levels of CEA in ABPA people had an increasing trend (which was more than general population values).

However, the present study has two important limitations. Firstly, this was a retrospective single-center study with a relatively small sample size. Secondly, we only included one patient who underwent biopsy, and it is more appropriate to study serum samples of ABPA patients labeled with both CEA and eosinophils. Therefore, further large-scale prospective study should be conducted to verify the preliminary results of the present study.

## Conclusion

In summary, the serum CEA levels were shown to be elevated relatively in ABPA patients. However, the elevated serum CEA level can be normalized after treatment. Increased levels of CEA in ABPA patients might be positively correlated with eosinophil levels, and eosinophils can serve as CEA-secreting cells in patients with ABPA.

## Abbreviations

ABPA: Allergic bronchopulmonary aspergillosis
CEA: Carcinoembryonic antigen
EBUS-TBLB: Endobronchial ultrasound-guided transbronchial lung biopsy aspiration
AIGA: Acquired idiopathic generalized anhidrosis
NETs: Neutrophil extracellular traps
ELISA: Enzyme-linked immunosorbent assay
IL-5: Interleukin-5
MBP: Myelin basic protein
H&E: Hematoxylin and eosin
BSA: Bovine serum albumin
PBS: Phosphate-buffered saline
DAPI: 4′,6-Diamidino-2-phenylindole
ISHAM: The International Society for Human and Animal Mycology
PET-CT: Positron emission tomography-computed tomography
