# Author Correction: Eosinophils may serve as CEA-secreting cells for allergic bronchopulmonary aspergillosis (ABPA) patients

**DOI:** 10.1038/s41598-021-89003-y

**Published:** 2021-05-06

**Authors:** Yanfei Yang, Gao qiqi, Yangyi Jin, Mengdie Qi, Guohua Lu, HequanLi

**Affiliations:** 1 The First Affiliated Hospital of Zhejiang University School of Medicine, 79 Qingchun Road, Hangzhou, 310003 Zhejiang China; 2 Hangzhou TCM Hospital Affiliated to Zhejiang Chinese Medical University, Hangzhou, Zhejiang China; 3 Tongde Hospital of Zhejiang Province, Hangzhou, Zhejiang China

Correction to: *Scientific Reports* 10.1038/s41598-021-83470-z, published online 17 February 2021

In the original version of this Article, Affiliations 1 and 2 were not listed in the correct order. The correct affiliations are listed below:

Affiliation 1:

The First Affiliated Hospital of Zhejiang University School of Medicine, 79 Qingchun Road, Hangzhou 310003, Zhejiang, China.

Affiliation 2:

Hangzhou TCM Hospital Affiliated to Zhejiang Chinese Medical University, Hangzhou, Zhejiang, China.

These errors have now been corrected in the PDF and HTML versions of the Article.

